# Survival of Patients with Oligometastatic Pancreatic Ductal Adenocarcinoma Treated with Combined Modality Treatment Including Surgical Resection: A Pilot Study

**DOI:** 10.1089/pancan.2018.0011

**Published:** 2018-11-01

**Authors:** Pujan Kandel, Michael B. Wallace, John Stauffer, Candice Bolan, Massimo Raimondo, Timothy A. Woodward, Victoria Gomez, Ashton W. Ritter, Horacio Asbun, Kabir Mody

**Affiliations:** ^1^ Department of Gastroenterology and Hepatology, Mayo Clinic, Jacksonville, Florida.; ^2^ Department of Surgery, Mayo Clinic, Jacksonville, Florida.; ^3^ Department of Radiology, Mayo Clinic, Jacksonville, Florida.; ^4^ Department of Hematology and Oncology, Mayo Clinic, Jacksonville, Florida.

**Keywords:** ablation, metastasectomy, overall survival, pancreaticoduodenectomy, pancreatic cancer

## Abstract

**Purpose:** To evaluate the overall survival of patients with oligometastatic pancreatic ductal adenocarcinoma (PDAC; metastatic tumor <4 cm, ≤2 metastatic tumors total) receiving neoadjuvant therapy, metastasectomy and/or ablation, and primary tumor resection.

**Methods:** We performed a case–control study from January 2005 to December 2015. Patients who underwent curative-intent surgery combined modality therapy (M1 surgery group; 6 [14%], tumor [T]3, node [N]1, and oligo-metastases [M]1) were matched 1 to 3 based on TN stage with two control groups (M0 surgery and M1 no surgery). The M0 surgery group (18 [43%], T3, N1, and M0) included patients without metastases who underwent resection. The M1 no surgery group (18 [43%], T3, N1, and M1) included patients with metastatic PDAC who received palliative chemotherapy without surgical resection.

**Results:** Median overall survival in the M1 surgery, M0 surgery, and M1 no surgery groups was 2.7 years (95% confidence interval [CI], 0.71–3.69), 2.02 years (95% CI, 0.98–3.05), and 0.98 years (95% CI, 0.55–1.25), respectively. Eastern Cooperative Oncology Group (ECOG) status was associated with survival (*p* = 0.01) after univariate analysis. After adjusting for ECOG status, multivariate analysis showed M1 surgery patients had improved survival compared with M1 no surgery patients and similar survival to M0 surgery patients.

**Conclusion:** Multimodal therapy benefitted our M1 surgery patients. A larger, prospective study of this multidisciplinary management strategy is currently under way.

## Introduction

Pancreatic cancer is the third leading cause of cancer-related death in United States, and it is anticipated to be the second most common cause by 2020.^1,2^ The 5-year median survival is 8%.^3^ Even though the cancer-related deaths associated with most cancers have declined over the past decade, the rate of death associated with pancreatic cancer has shown only slight improvement in overall survival.^3,4^ The shorter survival period and poorer outcomes may be due to the aggressive nature of this cancer, its late-stage presentation, and its tendency to develop early metastases. Surgery is one of the treatment options for pancreatic ductal adenocarcinoma (PDAC); however, only 15–20% of patients are eligible for curative intent surgery at the time of diagnosis.^5–8^

Patients with metastatic disease portend a worse survival and have traditionally been considered as having unresectable disease according to the National Comprehensive Cancer Network (NCCN) treatment guidelines.^9^ About 50% of the patients diagnosed with pancreas cancer have metastatic disease at the time of diagnosis.^10^ In addition, most patients will ultimately develop metastatic disease, even if they are diagnosed and treated in earlier stages of the disease. Metastases are most commonly seen in the liver, followed by the lungs, abdominal lymph nodes, peritoneum/omentum, and adrenal glands.^11–13^

There have been improvements in preoperative imaging technologies for early diagnosis of metastatic diseases, including pancreas-specific computed tomography (CT), magnetic resonance imaging (MRI), and endoscopic ultrasound (EUS).^2^ According to the U.S. population-based study, Surveillance Epidemiology and End Results database, preoperative evaluation with EUS is associated with increased survival in pancreatic cancer, likely attributed to early curative intent surgery and chemoradiation.^14^

However, in the setting of metastatic pancreas cancer, data are limited on surgical treatment of pancreatic cancer in the setting of metastatic disease, and resection is discouraged. Although, hepatic resection has been well established in other gastrointestinal malignancies such as colorectal cancer and neuroendocrine tumors with liver metastases,^15,16^ metastasectomies for PDAC remains highly controversial, and when it is applied indiscriminately to any patient with metastatic disease, it may not be of benefit. Nevertheless, newer treatments, such as neoadjuvant chemotherapy, radiofrequency ablation (RFA), simultaneous metastasectomy, and pancreatectomy could theoretically improve survival in a highly selected group of patients.

Available evidence from the literature provides little information about the guidelines of extended resections in PDAC, which has made it difficult to reach an objective conclusion regarding the outcomes following extended pancreaticoduodenectomy (PD). However, results from the largest series of metastasectomy in oligometastatic PDAC by Niesen et al.^17^ have reported favorable outcome and survival in a highly select group of patients following pancreatic surgery with concurrent resectable metastasis. The main objective of our study was to evaluate the overall survival and outcomes of an extremely select group of patients with PDAC with oligometastases treated with neoadjuvant chemotherapy, metastasectomy and/or RFA, and primary tumor resection.

## Materials and Methods

### Patient population

Patients with PDAC who were evaluated and treated at Mayo Clinic in Jacksonville, Florida, between January 2005 and December 2015, were eligible for study inclusion. Patients were also included if they had neoadjuvant/adjuvant chemotherapy or chemoradiation, palliative chemotherapy, metastasectomy (wedge resection or segmentectomy of liver), curative-intent pancreatic surgery, and oligometastatic disease (liver and lung) without evidence of other distance metastases. Patients were excluded if they had multiple-site metastases, periampullary cancer, neuroendocrine tumors, or cholangiocarcinoma. The Mayo Clinic Institutional Review Board approved the study protocol.

### Study design

We conducted a retrospective, case–control study at a high-volume surgical center. We adopted tumor resectability criteria according to NCCN guidelines, and staging information was documented using the tumor-node-metastasis (TNM) nomenclature from American Joint Committee on Cancer (AJCC). Preoperative imaging (EUS, MRI, and CT) was used as a baseline for staging and tumor resectability of pancreatic cancer. Patients with oligometastatic PDAC (≤2 metastatic tumors total in liver or lung, each <4 cm) who underwent neoadjuvant chemotherapy (FOLFIRINOX or Gemcitabine/nab-Paclitaxel), primary tumor resection, and metastasectomy and/or RFA (combined modality therapy) of metastatic disease (M1 surgery group) were compared with two control groups (M0 surgery and M1 no surgery). Cases (T3, N1, and oligo-M1) were matched at a 1:3 ratio based on TN stage with two control groups (Table 1). The M0 surgery (T3, N1, and M0) control group included patients without metastases who underwent resection. The M1 no surgery control group (T3, N1, and M1) included patients who did not undergo surgical resection, but completed palliative chemotherapy instead. All patients were presented at a multidisciplinary pancreas board meeting where management decisions were discussed.

**Table 1.** **Summary Demographics**

Characteristics	M1 surgery group (*n* = 6)	M0 surgery group (*n* = 18)	M1 no surgery group (*n* = 18)	*p*-Value
Age (years), median (range)	64 (60–71)	64 (44–80)	65 (56–91)	0.9^*^
≤65 years, *n* (%)	4 (67)	9 (50)	9 (50)	
>65 years, *n* (%)	2 (33)	9 (50)	9 (50)	
BMI, median (range)	29 (19–34)	25 (20–36)	25 (19–41)	0.4^*^
Sex, *n* (%)				
Male	6 (100)	5 (28)	10 (56)	0.02^**^
Female	0 (0)	13 (72)	8 (44)	
Race, *n* (%)				
White	6 (100)	17 (94)	18 (100)	
African American	0 (0)	1 (6)	0 (0)	
ECOG (0–1), median (range)	0 (0–1)	0 (0–1)	0 (0–1)	1.0^*^
ECOG-0, *n* (%)	5 (83)	15 (83)	15 (83)	
ECOG-1, *n* (%)	1 (17)	3 (17)	3 (17)	
Tumor location, *n* (%)				
Head	3 (50)	14 (79)	13 (72)	0.1^**^
Body and tail	3 (50)	4 (21)	5 (28)	
Tumor stage, *n* (%)				
T3	6 (100)	18 (100)	18 (100)	
Nodal status, *n* (%)				
N1	6 (100)	18 (100)	18 (100)	
Tumor resectability (at preoperative imaging), *n* (%)				1.0^**^
Resectable	3 (50)	9 (50)	9 (50)	
Borderline resectable	3 (50)	9 (50)	9 (50)	
Margins, *n* (%)				
R0	5 (83)	16 (89)	NA	
R1	1 (17)	2 (11)	NA	
Known metastatic site, *n* (%)				
Liver	4 (67)	NA	14 (78)	
Lung	2 (33)	NA	4 (22)	
Neoadjuvant treatment (%)				
Yes	6 (100)	8 (44)	NA	
% Chemo alone	5 (83)	4 (22)	NA	
% Chemo RT	1 (17)	4 (22)	NA	
CA-199 at diagnosis, (IU/L)				
Median (range)	210 (1–3388)	109 (4–1510)	691 (72–141,031)	0.009^*^
Metastatic disease treatment, *n* (%)				
Hepatic resection only (liver metastases)	1 (17)	NA	NA	
Hepatic resection and RFA (liver metastases)	2 (33)	NA	NA	
RFA only (lung metastases)	2 (33)	NA	NA	
Radioembolization only (liver metastases)	1 (17)	NA	NA	
Adjuvant/palliative therapy, *n* (%)	6 (100)	18 (100)	18 (100)	
% Adjuvant chemotherapy alone	5 (100)	4 (22)	18 (100)	
% Chemotherapy RT	1 (17)	14 (78)	0 (0)	
Accrual survival (95% CI) no. at risk				
1 Years	83% (36%–97%) 6	72% (53%–91%) 15	47% (26%–70%) 9	
2 Years	62% (21%–90%) 3	44% (23%–66%) 11	7% (1%–37%) 1	
3 Years	—	44 (23%–66%) 11	—	
5 Years	—	37% (17%–61%) 5	—	

^*^ Kruskal–Wallis test.

^**^ Fischer's exact test.

BMI, body mass index; CI, confidence interval; ECOG, Eastern Cooperative Oncology Group; M0 surgery group, no metastatic disease with surgical treatment; M1 no surgery group, metastatic disease with no surgical treatment; M1 surgery group, metastatic disease with surgical treatment; N1, lymph node positive; NA, not applicable; RFA, radio frequency ablation; RT, radiotherapy; T3, stage three.

### Statistical analysis

Statistical analysis was carried out using JMP (v10; SAS Institute Inc., Cary, NC) software. Baseline demographics are summarized with counts and percentages for categorical variables and as medians (95% confidence interval [CI]) for continuous variables. Because of the small sample size and non-normal distributions, the differences between the groups were compared using nonparametric statistical methods. The Fischer exact test was used to compare categorical variables and the Kruskal–Wallis test was used for continuous variables. Two-sided *p*-values <0.05 were considered statistically significant.

Survival and follow-up were calculated from the time of diagnosis to the date of death or last follow-up. Cumulative survival among the three groups was analyzed using the nonparametric product limit Kaplan–Meier method. Differences in survival among the groups were assessed using the log-rank test. To determine the prognostic factors associated with survival, univariate and multivariate analysis were performed using Cox proportional hazards model where applicable. A pair-wise comparison was conducted to examine the differences between the groups. Sample size was based on all available metastatic PDAC surgical cases at Mayo Clinic in Jacksonville, Florida. Three controls were matched to each case to maximize power. Addition of higher control: case ratios have minimal additional gains in power.^18^

## Results

A total number of 728 patients with PDAC were seen at Mayo Clinic, Jacksonville during this period. Of this 42 patients were included in this study, and 6 (14%) in M1 surgery group were matched for tumor resectability and TN stage with 18 (42%) in the M0 surgery group and 18 (42%) in the M1 no surgery group. Baseline demographics and clinicopathologic characteristics were comparable between the groups, apart from differences in sex (*p* = 0.02) and CA19-9 at diagnosis (*p* = 0.009; Table 1). At the time of diagnosis, all patients were AJCC/TNM stage T3 and N1, but varied in M stages. From baseline preoperative imaging in all three groups, 50% were resectable and 50% were borderline per NCCN treatment guidelines. Four patients (67%) in the M1 surgery group had liver metastases and 2 (33%) had lung, while in the M1 no surgery group, 14 (78%) had liver and 4 (22%) lung. In the M1 surgery group, the primary tumor surgery consisted of hepatic resection for 1 (17%) patient, RFA for 2 (33%), radioembolization for 1 (17%), and hepatic resection plus RFA for 2 (33%; Table 1). R0 resection was achieved in 5 (83%) patients in the M1 surgery group versus 16 (89%) in the M0 surgery group. All 42 patients received either adjuvant, palliative treatment, or both. However, while all 6 (100%) patients received neoadjuvant treatment in the M1 surgery group only 8 (43%) received it in the M0 surgery group.

### Survival analysis

**FIG. 1.**
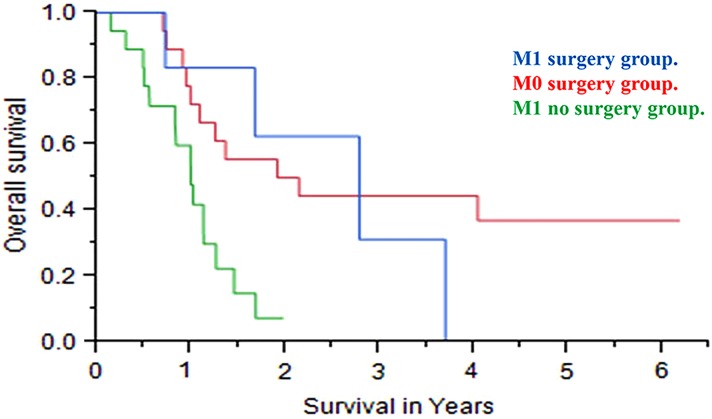
A Kaplan–Meier survival curve comparing survival in the three groups.

Overall median survival in the M1 surgery, M0 surgery, and M1 no surgery groups were 2.7 years (95% CI, 0.71–3.69), 2.02 years (95% CI, 0.98–3.05), and 0.98 years (95% CI, 0.55–1.25), respectively (Fig. 1). Overall median survival for the M1 surgery group was significantly better than in the M1 no surgery group (2.7 vs. 0.98 years, *p* = 0.01), but was similar to that of the median M0 surgery group (2.7 vs. 2.02 years, *p* = 0.6; Table 2). To further explore other factors associated with survival, we performed univariate and multivariate analysis. Only the Eastern Cooperative Oncology Group (ECOG) status was positively associated with survival after univariate analysis (Table 3). In the multivariate Cox proportional hazards model, and after adjusting for ECOG status, the M1 no surgery group had an increased risk of death compared with the M1 surgery group (5.1 [95% CI, 1.37–32.90], *p* = 0.01). There was no statistical difference in survival between the M1 surgery group and the M0 surgery group (1.7 [95% CI, 0.45–5.75], *p* = 0.3) (Table 4).

**Table 2.** **Univariate Analysis Comparing 3 Groups**

Groups comparison	Median overall survival in years (95% CI)	Log rank (*p*-value)
M1 surgery group	2.77 years (0.71–3.69)	0.6
M0 surgery group	2.02 years (0.98–3.05)	
M1 no surgery group	0.98 years (0.55–1.25)	0.01
M1 surgery group	2.77 years (0.71–3.69)	
M0 surgery group	2.02 years (0.98–3.05)	0.005
M1 no surgery group	0.98 years (0.55–1.25)	

**Table 3.** **Univariate Cox Regression Analysis for Predictors of Death**

Variables	HR (95% CI)	*p*-Value
Age (ref = ≤65 years)	1.5 (0.77–3.32)	0.2
Sex (ref = female)	1.6 (0.81–3.57)	0.1
BMI (ref = ≤25)	1.03 (0.48–2.12)	0.9
Margins, R1 (ref = R0)	1.6 (0.36–5.31)	0.4
ECOG (ref = 1)	0.2 (0.09–0.61)	0.004
CA 19-9 IU/L (ref = ≤200 IU/L)	1.2 (0.55–2.69)	0.6

HR, hazard ratio.

**Table 4.** **Type of Surgery and Survival of M1 Surgery Patients**

No	Primary tumor	Site of metastases	Treatment of metastatic disease	Type of surgery	Survival (years)	Status
1	Pancreatic head cancer	Lung	RFA	Laparoscopic Whipple with portal vein reconstruction	3.7	Dead
2	Pancreatic head cancer	Liver	RFA plus liver resection	Laparoscopic pylorus preserving pancreaticoduodenectomy with liver resection	1.9	Alive
3	Pancreatic head cancer	Liver	RFA plus liver resection	Pylorus preserving pancreaticoduodenectomy with liver resection	1.2	Alive
4	Pancreas body and tail cancer	Liver	Liver resection	Laparoscopic distal pancreatectomy with hepatic resection with splenectomy	2.8	Dead
5	Pancreas body and tail cancer	Lung	RFA	Laparoscopic distal pancreatectomy with splenectomy.	0.8	Dead
6	Pancreas body and tail cancer	Liver	Radioembolization of liver metastases	Laparoscopic distal pancreatectomy with splenectomy	1.6	Dead

M1 surgery patients: patients with metastatic disease who underwent surgical treatment.

RFA, radiofrequency ablation.

## Discussion

Pancreatic cancer has a poor prognosis. Surgical technique and outcomes following surgical resection has evolved over the years,^19^ but controversy exists surrounding the concept of metastasectomy in the management of metastatic pancreas cancer.

Our study explored the survival benefits of definitive metastatic disease management in select patients with metastatic pancreas cancer, treated at a high-volume, tertiary care cancer center. The patients included in our study had high performance status, two or fewer liver or lung metastases, and R0 resection following surgical resection. Our results suggest that combined modality treatment (neoadjuvant chemotherapy, metastasectomy and/or RFA, and surgical resection) for oligometastatic PDAC can be performed in a highly selective group of patients, with survival similar to TN-stage matched patients without metastases and improved survival compared with nonresected patients.

Data from other studies exploring this approach indicate that metastasectomy can be performed safely and with improved survival.^17,20–23^ Adam et al.^24^ demonstrated a median survival of about 20 months in patients who underwent concurrent hepatic and primary pancreatic tumor resection. The overall five year survival rate was 25%. Shrikhande et al.^25^ reported that 11 PDAC patients with liver metastases who underwent concurrent liver and pancreatic resection had a longer median survival than patients who had undergone exploratory surgery without resection (11.4 vs. 5.9 months, *p* = 0.03). Results from a large cohort study by Niesen et al.^17^ reported that the overall median survival after M1 resection in 128 patients (85 liver and 43 inguinal lymph node) was significantly better than exploratory surgery alone (14 months vs. 8.6 months). The number of liver resections was 1 or 2 in 76% of patients and more than 3 in 24%. Additionally, the M1 resection (liver) group had better 5-year overall survival (9.7% vs. 0%). A systematic review by Michalski et al.^26^ of 103 patients from three case reports and 18 studies involving fewer than 10 patients with one or two liver metastases undergoing synchronous hepatic resection with PD found an overall survival comparable with patients without metastatic disease undergoing PD. Recently published retrospective data from multicenter studies have also shown that patients who had undergone simultaneous pancreatic and liver resection had significantly better overall survival than patients undergoing exploratory surgery without tumor resection (14 months vs. 8 months, *p* < 0.001).^27^ We think best approach would be not to proceed with surgery alone but to include systemic treatment with modern targeted chemotherapy.

With advances in surgical techniques and perioperative care, the overall perioperative morbidity and mortality of pancreatic surgery has decreased. Metastasectomy can be performed in some patients, but the selection of patients is critical to any pursuit of surgical management. Our study and one other suggest the following patient selection criteria: a good performance status; two or fewer metastatic tumors, no more than 4 cm in diameter; primary pancreatic tumor with achievable R0 resection; resectable metastatic disease; and evidence of response to neoadjuvant chemotherapy, chemoradiation, or both by RESIST criteria.^28^ However, in our study treatment effects were evaluated by an abdominal multiphasic pancreatic protocol CT or MRI. Response was defined simply as a lack of progression of disease, especially development of new sites of disease, while on chemotherapy. Additionally, our disease response definition included our surgery team's assessment that resection was technically feasible. Response Evaluation Criteria in Solid Tumors (RECIST) were not routinely used for response assessment for patients cared for in routine clinical practice, off clinical trial.

Our study has many limitations. First, the sample size is small; therefore, the study had inadequate statistical power to clearly distinguish whether the observed similar outcomes compared with the M0 surgery group are truly similar. Second, the retrospective nature of our study does not allow us to fully exclude biases in selection of patients, although we attempted to minimize this by carefully matching tumor and nodal stage. However, other important biological differences between tumors and patients could not be matched and were controlled in multivariable modeling. Another limitation is that the study was single institution based and only patients who tolerated neoadjuvant chemotherapy and remained fit for surgery were included.

We conducted a retrospective study of multimodal, curative-intent management of oligometastatic pancreatic cancer in a highly selective group of patients treated at a high-volume, tertiary-care cancer center. These patients may achieve significantly better survival with multimodal therapy that includes chemotherapy, metastasectomy and/or RFA, and primary tumor resection. A larger, prospective study of this multidisciplinary management strategy is currently under way.

## Abbreviations Used

AJCC: American Joint Committee on Cancer
BMI: body mass index
CI: confidence interval
CT: computed tomography
ECOG: Eastern Cooperative Oncology Group
EUS: endoscopic ultrasound
HR: hazard ratio
MRI: magnetic resonance imaging
NA: not applicable
NCCN: National Comprehensive Cancer Network
PD: pancreaticoduodenectomy
PDAC: pancreatic ductal adenocarcinoma
RFA: radiofrequency ablation
RT: radiotherapy
TNM: tumor-node-metastasis
